# Use of Slow-Release Injectable Moxidectin for Treatment of *Dirofilaria immitis* Infection During Pregnancy

**DOI:** 10.3389/fvets.2019.00440

**Published:** 2020-01-28

**Authors:** Bruno Alberigi, Celeste da Silva Freitas de Souza, Julio Israel Fernandes, Alexandre Merlo, Norma Labarthe

**Affiliations:** ^1^Programa de Pós-Graduação em Medicina Veterinária, Universidade Federal Rural do Rio de Janeiro, Rio de Janeiro, Brazil; ^2^Laboratório de Imunomodulação e Protozoologia, Instituto Oswaldo Cruz, Fundação Oswaldo Cruz, Rio de Janeiro, Brazil; ^3^Departamento de Medicina e Cirurgia Veterinária, Instituto de Veterinária, Universidade Federal Rural do Rio de Janeiro, Rio de Janeiro, Brazil; ^4^Technical Services for Companion Animals, Zoetis, São Paulo, Brazil; ^5^Programa de Pós-Graduação em Bioética, Ética Aplicada e Saúde Coletiva. Escola Nacional de Saúde Pública, Fundação Oswaldo Cruz, Rio de Janeiro, Brazil

**Keywords:** heartworm, microfilariae, macrocyclic lactone, doxycycline, vector-borne disease

## Abstract

Canine heartworm disease is a life-threatening disease caused by *Dirofilaria immitis* and is prevalent in Brazil. The standard drug for its treatment, melarsomine dihydrochloride, is a fast-killing organic arsenical chemotherapeutic agent not approved in Brazil. Therefore, an alternative strategy, such as macrocyclic lactone in combination with a tetracycline antibiotic, has to be used. The alternative method is a long-term therapy that could lead to compliance issues during treatment. The aim of this case report is to present a preliminary assessment on the efficacy and safety of an off-label biannual administration of slow-release moxidectin (0.5 mg/kg every 6 months), which is formulated for annual administration (0.5 mg/kg annually). This overdose was chosen to test if moxidectin serum levels could be maintained high enough to harm the worms. It was administered to a 4-year-old female dog in combination with a 30-day doxycycline course. The second dose of moxidectin was administered approximately a week before she gave birth to three healthy puppies. Microfilariae were not detected on day 180 of treatment. Serological tests showed that the worms were eliminated, as two negative antigen tests were obtained 6 months apart (at day 180 and day 360 of treatment). Therefore, the off-label biannual use of moxidectin in combination with doxycycline was effective in eliminating *D. immitis* in 360 days and was harmless for the pregnant dog and her offspring, suggesting that this strategy is promising. Although these results are encouraging, further studies are needed to confirm safety and efficacy issues.

## Background

Canine heartworm disease is life threatening when left untreated (1, 2); therefore, treatment must be administered even under adverse conditions. The objective of the treatment must be to eliminate *D. immitis* in all stages as quickly as possible to improve the animal's clinical condition (2).

There are 2 distinct drug-based methods to eliminate the infection. Veterinarians must consider the animal's clinical conditions, stage of the disease, and treatment risk prior to deciding the treatment (2–4). Internationally recommended treatment is the fast-acting organic arsenical compound melarsomine dihydrochloride (2, 5–7), despite being unavailable in many countries (7), and the risk that it poses to severely ill dogs (2, 8). The reported advantage of this treatment is the speed at which it kills adult worms and prevents the heart and lungs disease from progressing (2, 7). The other method is the long-term use of macrocyclic lactone in combination with a tetracycline antibiotic. The antibiotic with best therapeutic results is doxycycline (9) and the macrocyclic lactones reported as efficacious are oral ivermectin (10–12), topical moxidectin (13, 14), and double annual dose of injectable moxidectin (15). This long-term treatment is also called “alternative treatment,” “slow kill,” “soft kill,” “doxy-moxi,” or “moxi-doxy” and is recommended to be used when the arsenical drug is unavailable or when fast kill is contraindicated (2, 7). The injectable moxidectin is an extended-release injectable formulation that can be used in breeding animals and a 5X safety margin has been shown (16).

## Case Presentation

A 4-year-old (estimated) Pit Bull cross intact female (25 kg) was presented for routine evaluation after it was adopted from the streets. The results showed that she had asymptomatic *D. immitis* infection as indicated by the presence of microfilariae in Knott's modified test (17) and a positive *D. immitis* antigen test, although seronegative for *Anaplasma* spp., *Ehrlichia* spp., and *Borrelia burgdorferi* (SNAP^®^4DX Plus^®^, IDEXX Laboratories Inc., Westbrook, ME, USA). She was seronegative for *Leishmania infantum* (TR DPP^®^ canine visceral leishmaniasis, Bio-Manguinhos, Brazil). The routine physical examination showed no clinical signs of the disease and the hematological and urine examination results were normal.

**Figure 1:**
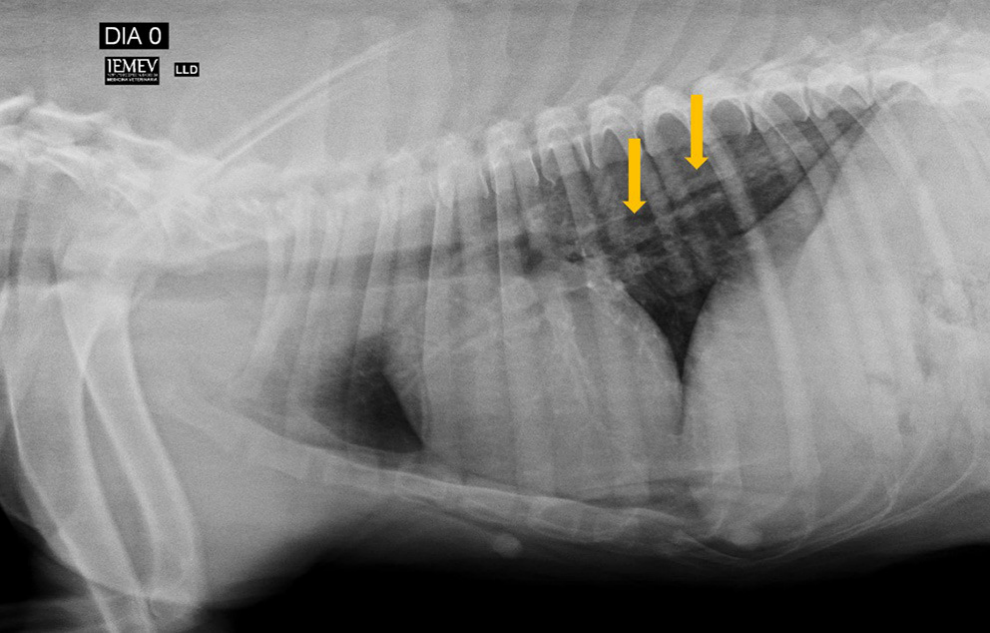
Right lateral position showing increased pulmonary radiodensity, especially characterized by a diffusely distributed bronchial pattern (arrows).

**Figure 2:**
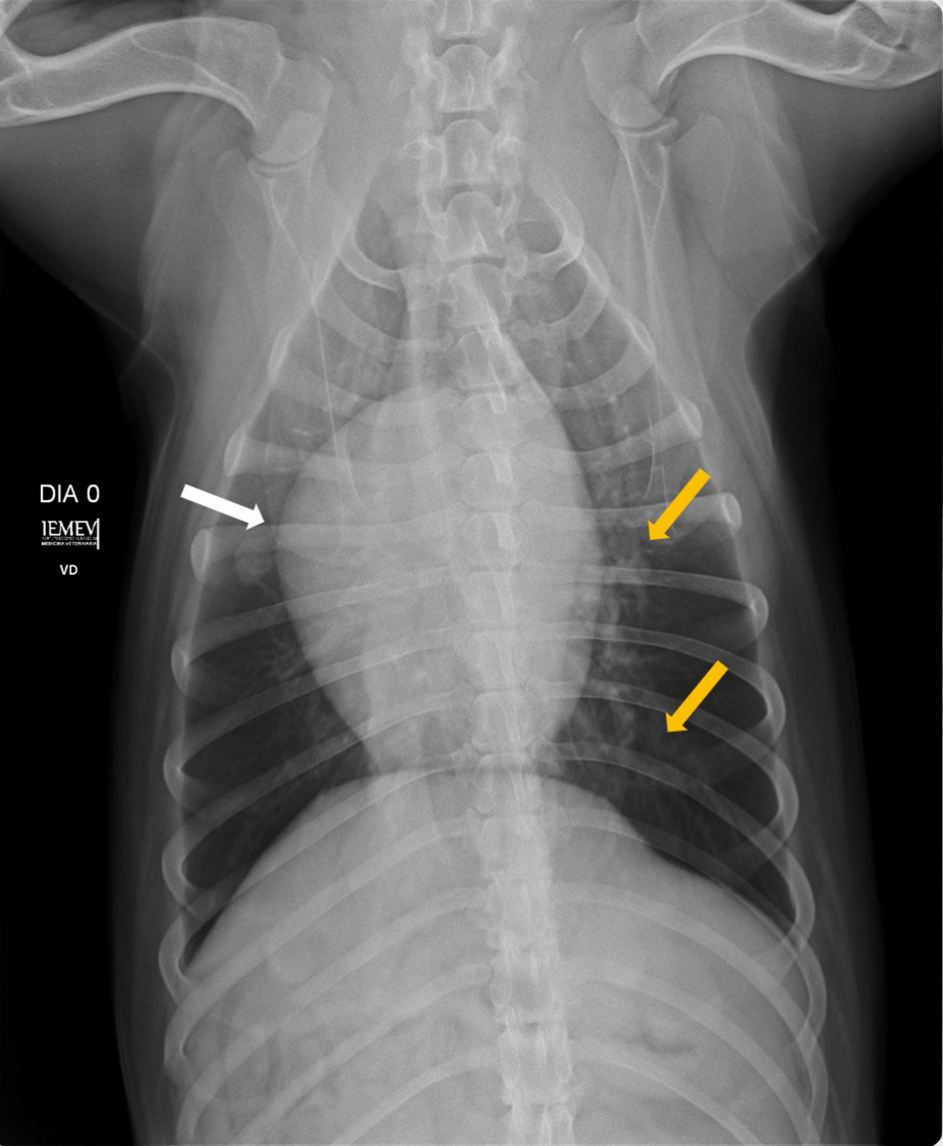
Ventrodorsal position showing right atrioventricular enlargement (white arrow) and diffusely distributed bronchial pattern (yellow arrows).

The heartworm pre-treatment tests included blood work (blood urea nitrogen test, creatinine, alanine transaminase, and alkaline phosphatase levels and complete blood count), Doppler echocardiogram and chest X-rays. The blood work and Doppler echocardiogram were normal; however, the X-rays showed mild right atrioventricular enlargement and mild increase in interstitial and bronchial pulmonary pattern (Figures 1, 2).

Since the organic arsenical drug is unavailable in Brazil, the treatment option was the use of slow-release injectable moxidectin (ProHeart^®^ SR-12, Zoetis, Campinas, Brazil) biannually (0.5 mg/kg) instead of annually in combination with 30 days of doxycycline (Doxifin^®^, Ourofino, Cravinhos, Brazil) (10 mg/kg/BID). The owner was duly informed and clarified about the off-label alternative treatment and after her formal consent the administration of both drugs was initiated on the same day.

When the dog was presented for evaluation 6 months following the first moxidectin injection it was informed that she could be pregnant as she had been in heat approximately 30 days before the visit and she lived with an intact male dog. Abdominal ultrasound was performed, and pregnancy was confirmed. Therefore, chest X-rays for lungs evaluation were precluded. All the other pre-treatment examinations were repeated, and the results were within the reference range. No microfilariae were detected by Knott's modified test and *D. immitis* antigen test was negative. Since a *D. immitis*-infected dog receiving alternative treatment must present microfilariae and *D. immitis* antigen test negative results 6 months apart to confirm the elimination of the parasite, a second moxidectin injection (0.5 mg/kg) was administered 6 months after the first and the animal was kept under observation. Within 1 week following the second injection, the animal gave birth to three healthy puppies.

Blood work, Doppler echocardiogram, and chest X-rays were unchanged when she was presented 6 months after the second moxidectin dose and serological tests with the heat pre-treatment sample (18) confirmed that heartworm had been successfully eliminated. Since the animal was free of infection, the owner was advised to continue with the moxidectin injections annually.

## Discussion

Although the exact time period the alternative method took to eliminate the infection is unknown, a negative *D. immitis* antigen test result was obtained at day 180 of treatment. When this is compared to the time needed by the recommended organic arsenical drug protocol to eliminate the infection (2), the alternative method took no more than 60 days longer. In addition, the elimination of microfilariae suggests that the alternative method is inoffensive to macrocyclic lactone resistance development. Therefore, alternative methods using moxidectin in combination with doxycycline are valid treatment options (9, 11, 13–15), particularly when melarsomine dihydrochloride is unavailable or contraindicated.

Even though this is a single case report, it adds evidence to show the safety of the off-label use of the annual slow-release injectable moxidectin formulation biannually as expected (19). Even more important is the fact that the use of a double dose of the slow release injectable moxidectin formulation (0.5 mg/kg every 6 months) caused no side effect whatsoever on the mother or her offspring.

The off-label biannual use of the slow-release injectable moxidectin (0.5 mg/kg every 6 months) in combination with doxycycline was efficacious in eliminating *D. immitis* infection. This treatment was clinically safe for the animal even during pregnancy and for her off-spring. In an overall view it can be suggested that veterinarians may use it for controlling heartworm disease at their discretion, although further studies are needed.

## Data Availability Statement

All datasets generated for this study are included in the article/supplementary material.

## Ethics Statement

Written informed consent was obtained from the owner of the animal for the treatment and for the publication of this case report.

## Author Contributions

BA designed the treatment protocol, participated in the acquisition of data, interpretation of results, helped draft the manuscript, and was the primary veterinary practitioner for this case. CS performed the parasitological analysis and interpretation, and contributed to writing the manuscript. JF contributed to writing the manuscript. AM designed the treatment protocol and drafted the manuscript. NL designed the treatment protocol, assisted in interpretation of results, and helped draft the manuscript. All authors read and approved the final manuscript.

### Conflict of Interest

BA received the medication from Zoetis Brazil and from Ourofino Saúde Animal. AM is a current employee of Zoetis in Brazil. NL is a consultant for Bayer Animal Health, Boehringer Ingelheim Animal Health, Idexx Laboratories, and Zoetis in Brazil. The remaining authors declare that the research was conducted in the absence of any commercial or financial relationships that could be construed as a potential conflict of interest.
